# Levetiracetam versus fosphenytoin as a second-line treatment after diazepam for adult convulsive status epilepticus: a multicentre non-inferiority randomised control trial

**DOI:** 10.1136/jnnp-2022-329485

**Published:** 2022-10-07

**Authors:** Kensuke Nakamura, Aiki Marushima, Yuji Takahashi, Masaki Mochizuki, Akio Kimura, Yu Fukuda, Masahiro Asami, Hidetoshi Nakamoto, Satoshi Egawa, Junya Kaneko, Kyoko Unemoto, Yutaka Kondo, Chikara Yonekawa, Masatoshi Uchida, Eisei Hoshiyama, Takeshi Yamada, Kazushi Maruo, Eiichi Ishikawa, Yuji Matsumaru, Yoshiaki Inoue, IENE ECT with the LIFE study group

**Affiliations:** 1 Department of Emergency and Critical Care Medicine, Hitachi General Hospital, Hitachi, Ibaraki, Japan; 2 Department of Emergency and Critical Care Medicine, University of Tsukuba, Tsukuba, Ibaraki, Japan; 3 Division of Stroke prevention and treatment, Department of Neurosurgery, Faculty of Medicine, University of Tsukuba, Tsukuba, Ibaraki, Japan; 4 Department of Neurosurgery, Faculty of Medicine, University of Tsukuba, Tsukuba, Ibaraki, Japan; 5 Epilepsy Center, University of Tsukuba Hospital, Tsukuba, Ibaraki, Japan; 6 Department of Emergency Medicine and Critical Care, National Center for Global Health and Medicine, Toyama Shinjuku, Tokyo, Japan; 7 Department of Emergency Medicine, Teikyo University Hospital, Itabashi, Tokyo, Japan; 8 Neurointensive Care Unit, Neurosurgery, Stroke and Epilepsy Center, TMG Asaka Medical Center, Asaka, Saitama, Japan; 9 Department of Emergency and Critical Care Medicine, Nippon Medical School Tama Nagayama Hospital, Tama, Tokyo, Japan; 10 Department of Emergency and Critical Care Medicine, Juntendo University Urayasu Hospital, Urayasu, Chiba, Japan; 11 Department of Emergency and Critical Care Medicine, Jichi Medical University Hospital, Shimotsuke, Tochigi, Japan; 12 Emergency and Critical Care Medical Center, Dokkyo Medical University, Shimotsuga, Tochigi, Japan; 13 Tsukuba Clinical Research and Development Organization (T-CReDO), University of Tsukuba, Tsukuba, Ibaraki, Japan; 14 Department of Biostatistics, Faculty of Medicine, University of Tsukuba, Tsukuba, Ibaraki, Japan

**Keywords:** EPILEPSY, MEDICINE

## Abstract

**Objective:**

Status epilepticus (SE) is an emergency condition for which rapid and secured cessation is crucial. Although fosphenytoin (FPHT) is recommended as a second-line treatment, levetiracetam (LEV) reportedly has similar efficacy, but higher safety. Therefore, we herein compared LEV with FPHT in adult SE.

**Methods:**

We initiated a multicentre randomised control trial in emergency departments with adult patients with convulsive SE. Diazepam was initially administered, followed intravenously by FPHT at 22.5 mg/kg or LEV at 1000–3000 mg. The primary outcome was assigned as the seizure cessation rate within 30 min of the administration of the study drug.

**Results:**

A total of 176 adult patients with SE were enrolled (82 FPHT and 94 LEV), and 3 were excluded from the full analysis set. Seizure cessation rates within 30 min were 83.8% (67/80) in the FPHT group and 89.2% (83/93) in the LEV group. The difference in these rates was 5.5% (95% CI −4.7 to 15.7, p=0.29). The non-inferiority of LEV to FPHT was confirmed with p<0.001 by the Farrington-Manning test. No significant differences were observed in the seizure recurrence rate or intubation rate within 24 hours. Serious adverse events developed in three patients in the FPHT group and none in the LEV group (p=0.061).

**Conclusion:**

The efficacy of LEV was similar to that of FPHT for adult SE following the administration of diazepam. LEV may be recommended as a second-line treatment for SE along with phenytoin/FPHT.

**Trial registration number:**

jRCTs031190160.

**WHAT IS ALREADY KNOWN ON THIS TOPIC**

- Phenytoin/fosphenytoin are recommended as a second-line treatment for status epilepticus; however, these drugs are associated with serious adverse events. Levetiracetam is expected to be as effective, but with less serious adverse events because previous randomised control trials (RCTs) that compared them reported similar efficacies.

**WHAT THIS STUDY ADDS**

- There has yet to be a study with a positive result or non-inferiority designed RCT, particularly for adult status epilepticus. We herein conducted a multicentre non-inferiority designed RCT, in which adult patients with status epilepticus were randomised into levetiracetam and fosphenytoin groups as a second-line treatment after diazepam. The non-inferiority of levetiracetam to fosphenytoin was confirmed with less serious adverse events in the levetiracetam group.

**HOW THIS STUDY MIGHT AFFECT RESEARCH, PRACTICE OR POLICY**

- Levetiracetam may be used as a second-line treatment for adult status epilepticus. Each guideline and clinical practice for status epilepticus may add it as an alternative to phenytoin/fosphenytoin.

## Background

Status epilepticus (SE) is an emergency condition that is life-threatening with respiratory and circulatory system failure and may cause irreversible cerebral damage.^1^ Therefore, the rapid and secured cessation of seizures is crucial in addition to resuscitation.^2^ Potent gamma aminobutyric acid agonists, including benzodiazepines, are recommended as first-line treatments.^3 4^ However, other long-acting antiepileptic drugs (AEDs) are also required as second-line treatments for the complete cessation of SE and prevention of recurrence because benzodiazepines only act for short periods.^5^

Phenytoin is recommended as an effective second-line therapy for SE.^6^ Intravenous fosphenytoin (FPHT), the prodrug of phenytoin, is associated with fewer adverse events and is often used to treat SE.^7^ However, serious adverse events associated with the use of FPHT, such as hypotension, arrhythmia and allergic reactions, are similar to those of phenytoin in the treatment of SE, during which it is crucially important to maintain circulation and respiration.^8 9^ The risk of these adverse events is increased in elderly patients or those with cardiac disease.^10^

Levetiracetam (LEV), which primarily binds to synaptic vesicle protein 2A and regulates the release of neurotransmitters,^11^ is considered to be effective for SE with less serious adverse events.^12 13^ However, its use for SE is not covered by the national health insurance systems in many countries. Previous randomised control trials (RCTs) that compared intravenous LEV and intravenous phenytoin reported similar efficacies and serious adverse events.^14–19^ However, few RCTs have compared LEV with FPHT in adult patients, including the elderly. Moreover, a non-inferiority RCT has not yet been performed to examine the efficacy of intravenous LEV. Our nationwide database analysis showed the more frequent use of intravenous LEV than intravenous FPHT to treat adult SE despite the lack of coverage by the national health insurance system,^20^ and revealed the higher efficacy and safety of intravenous LEV.^21^

To establish intravenous LEV for the treatment of SE, we herein conducted a multicentre non-inferiority RCT, in which adult patients with convulsive SE transported to an emergency room were randomised into LEV and FPHT groups as a second-line treatment after the administration of diazepam, a typical benzodiazepine and their efficacies were compared. Since the participating facilities were recruited around Ibaraki in Japan, this study was entitled the *I*baraki *E*mergency room *NE*twork *E*pilepsy *C*ontrol *T*rial with *L*evetIracetam versus *F*osph*E*nytoine; IENE ECT with LIFE.

## Materials and methods

### Design

A multicentre, prospective and non-blinded RCT was conducted to compare the efficacy and safety of intravenous LEV and intravenous FPHT for the treatment of adult SE in the emergency room. The primary aim of the present study was to examine the non-inferiority of the efficacy of LEV to that of FPHT as a second-line treatment for SE after the administration of diazepam. The present study was conducted as a Japanese Association for Acute Medicine initiative study. It was registered at the Japan Registry of Clinical Trials (https://jrct.niph.go.jp/re/reports/detail/3358). The protocol employed was described in detail in a previous trial.^22^

### Participants, setting

Between 23 December 2019 and 31 March 2022, 176 consecutive patients with convulsive SE transported to nine emergency departments were enrolled. We finished the study when 176 patients were enrolled, irrespective of the patient number in each group. Since scheduled enrolment was achieved earlier, patient registration was stopped and the study was completed in September 2021.

The definition of SE is ‘continuous seizures longer than 5 min or discrete seizures longer than 2 min with intervening consciousness disturbance^5 23 24^; Japan Coma Scale II-30; the patient may only be aroused by repeated mechanical stimuli (then reverts to the previous state after the cessation of the stimulation)’.^25^ We enrolled patients with convulsive SE, in whom readily apparent convulsions were identified. Exclusion criteria were as follows: (1) younger than 20 years old, (2) previously recruited to the present study, (3) enrolment in the present study rejected by a proxy, (4) already intubated before treatment, (5) allergic to FPHT or LEV, (6) pregnancy, (7) epilepsy mimicker, (8) non-convulsive seizures when the study drugs were administered and (9) others judged to be ineligible by a physician. While physicians may exclude patients with criterion 9, we did not set the obvious exclusion criteria of cardiovascular/neurological/hepatic/metabolic disorders or already receiving the same medication.

Informed consent was obtained from a proxy before the study procedure. If no proxy was contactable, the study was conducted immediately without informed consent and researchers then obtained consent when the patient became alert or the proxy was found. Even if consent was obtained from a proxy, the researchers attempted to inform the patient after they become alert and then obtained consent. If enrolment was rejected, data from that patient were not used in analyses. If patients were unconscious and there was no proxy during hospitalisation, we included them in the analysis.

### Interventions

Resuscitation and stabilisation were simultaneously performed. Diazepam was intravenously administered at 1–20 mg. The physician selected the dose of diazepam to stop seizures. Following the intravenous administration of diazepam, electronic data capturing (EDC) (TXP Medical) was registered using a smartphone or personal computer, after which data were rapidly randomised and allocated to the FPHT and LEV groups. Block randomisation was performed using EDC, in which a random sequence was automatically generated for the two, four and eight participant units in each hospital. Either of the two, four or eight blocks was also randomly assigned. Therefore, stratification was performed only for the facilities.

In the FPHT group, FPHT at 22.5 mg/kg (phenytoin equivalent dose of 15 mg/kg) was intravenously administered in 100 mL of normal saline after diazepam at an administration rate not exceeding 3 mg/kg/min or 150 mg/min. In the LEV group, LEV at 1000–3000 mg was intravenously administered in 100 mL of normal saline after diazepam at an administration rate of 2–5 mg/kg/min. In both groups, height and body weight were estimated from body habitus, family information or patient records. All intervention medication doses were approved by the Japanese SE guidelines.^26^ After the cessation of seizures, electroencephalography (EEG) was performed as an option.

If convulsions were not stopped by these treatments, midazolam, propofol, thiopental or thiamylal was administered as a third-line treatment according to the Japanese guidelines.^26^ Other treatments, including intubation or intensive care, were not defined by the protocol. FPHT or LEV was randomised only for the first administration after diazepam and their subsequent administration was not regulated. While physicians were recommended to use the same study drug (FPHT/LEV) after the control of SE within 24 hours, they were permitted to administer the other study drug after the primary outcome evaluation where necessary.

### Outcomes

The primary outcome was the seizure cessation rate within 30 min of starting administration of the study drug. Seizure cessation in each patient was defined as the cessation of an apparent seizure 30 min after the administration of FPHT or LEV. Seizure cessation was not achieved when convulsions continued, convulsions reoccurred within 30 min, or a third-line treatment, described above, was introduced within 30 min.

Secondary outcomes were as follows: (1) the seizure recurrence rate within 24 hours, which was confirmed by an apparent seizure or non-convulsive seizure detected by EEG; (2) the serious adverse event rate throughout the observational period potentially induced by the study drugs, such as cardiac arrest, life-threatening arrhythmia, respiratory arrest and hypotension; and (3) the intubation rate within 24 hours.

Other observation items were as follows: (1) basic information on age, sex, height and body weight; (2) the type of SE; (3) seizure duration before treatment; (4) the cause of SE; (5) the modified Rankin Scale 7 days after admission; (6) the administered dose of diazepam and the time between intravenous diazepam and intravenous study drugs; (7) the administered dose of FPHT and LEV at loading and within 24 hours; (8) a previous history of liver disease; and (9) serum creatinine levels on admission.^27^

### Adverse events reporting, monitoring and interim analysis

On-site monitoring was performed at each hospital by monitors appointed by the monitoring committee. Central monitoring was also conducted. Adverse events were reported on medical records and EDC, with causal associations with intervention drugs, dates, severity, with/without any treatments and outcomes. Serious adverse events were immediately reported to the principal investigator, who then reported them to The Certified Review Board and Minister of Health, Labour and Welfare. The Certified Review Board had the power to stop the study when a marked difference was noted in safety based on reports of serious adverse events or safety monitoring. Other adverse events were reported on EDC. Spontaneous reporting was used over the course of the trial to non-systematically collect these adverse events. An efficacy interim analysis was not performed because this study was a non-inferiority RCT.

### Sample size estimation

The rate of effectiveness of each AED for SE was not assessed,^12^ particularly for ‘diazepam and FPHT’ and ‘diazepam and LEV’. Based on previous findings, the effectiveness of benzodiazepine alone was expected to be 50%–65%.^4 12 28 29^ In the present study, since outcomes were evaluated 30 min after the administration of the study drug, we estimated the efficacy of diazepam and FPHT for SE to be 65%.^4^ We then assigned a non-inferiority margin of an absolute difference of 20%, for which efficacy is clinically capable and that of diazepam and LEV will be higher than 45%, which was previously reported as the lowest efficacy rate.^4^ With a type I error (α=0.05) and type II error (β=0.2), we calculated the sample size as 176 patients.

### Statistical analysis

Statistical analyses were performed with a full analysis set (FAS) and safety analysis set (SAS). FAS was defined as all subjects without violations of the main eligibility criteria (selection and exclusion criteria) or conflicts with discontinuation and dropout criteria. SAS was defined as all subjects who received the study treatment. An efficacy analysis was performed with FAS. The safety analysis was conducted with SAS.

In the primary efficacy analysis, non-inferiority was examined using the Farrington-Manning test for efficacy differences from a non-inferiority margin of 20%. Differences in secondary outcomes were evaluated using χ^2^ tests. Regarding the other variables, continuous variables with a normal distribution were expressed as the mean±SD and compared using the Student’s t-test. Non-parametric continuous variables were expressed as medians (IQRs) and compared using the Mann-Whitney U test. Statistical analyses were conducted using SAS V.9.4 (SAS Institute, Cary, North Carolina, USA). P values <0.05 were considered to be significant.

## Results

**Figure 1:**
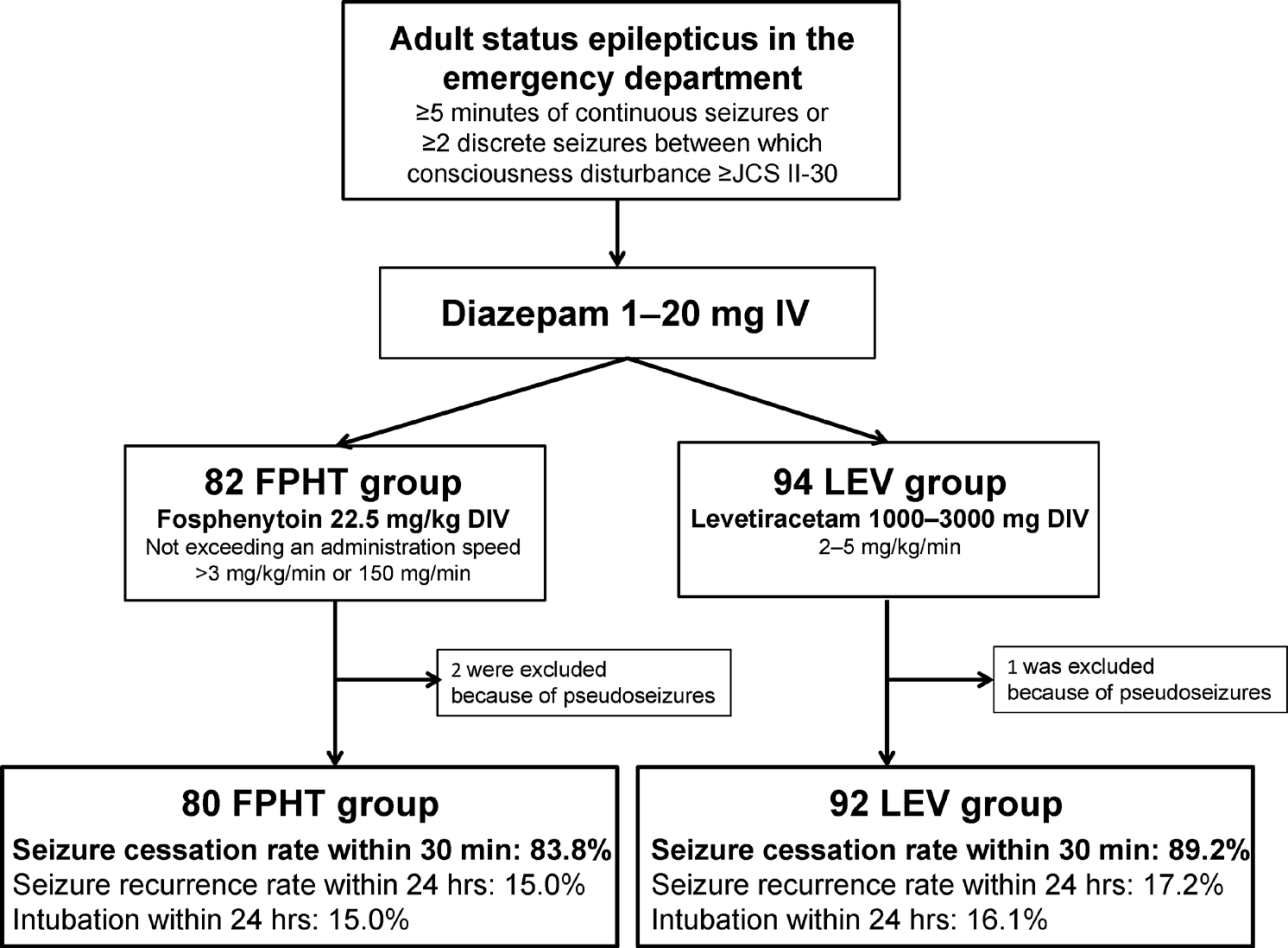
Study outline and outcomes. The study protocol was immediately performed on patients with status epilepticus who met the eligibility criteria in the emergency department. Registration was simultaneously conducted with the administration of diazepam and patients were randomised to the FPHT or LEV group. In both groups, diazepam was intravenously administered at 1–20 mg. In the 82 patients in the FPHT group, FPHT was intravenously administered at 22.5 mg/kg after diazepam at an administration rate not exceeding 3 mg/kg/min or 150 mg/min. In the 94 patients in the LEV group, LEV was intravenously administered at 1000–3000 mg after diazepam at an administration rate of 2–5 mg/kg/min. Two patients in the FPHT group and one in the LEV group were excluded because they were diagnosed with pseudoseizures. Seizure cessation rates within 30 min were 83.8% in the FPHT group and 89.2% in the LEV group. DIV, drip intravenous injection;FPHT, fosphenytoin; IV, intravenously; LEV, levetiracetam;JCS, Japan Coma Scale.

Among the 176 adult patients with convulsive SE enrolled during the study period, 82 were assigned to the FPHT group and 94 to the LEV group. The protocol was performed and completed on all patients. We did not obtain consent from 13 patients because they were unconscious and there was no proxy during hospitalisation, and we included them in the analysis according to the study design. No patient rejected enrolment after providing informed consent. Following considerations of safety, seven patients were administered 500 mg of LEV only, while one patient was administered 120 mg of FPHT only. Other protocol deviations were not observed. We included these eight patients in the analysis set. Three out of the 176 patients enrolled, 2 in the FPHT group and 1 in the LEV group, were diagnosed with pseudoseizures and removed from the analysis. Therefore, we included 176 patients in SAS and 173 patients in FAS after the exclusion of 3 dropout patients. The study outline and main outcomes are shown in figure 1.

Basic characteristics are shown in table 1. In the FPHT and LEV groups, mean ages were 65 and 67 years old, men accounted for 65.2% and 67.0%, mean heights were 163.0 cm and 161.5 cm, mean body weights were 60.3 kg and 57.9 kg and median serum creatinine levels on admission were 0.87 mg/dL and 0.86 mg/dL, respectively. Three patients in each group had a previous history of liver disease. The type of SE was mostly tonic clonic seizures; 71.3% in the FPHT group and 77.4% in the LEV group. The main cause of SE was a previous stroke, followed by idiopathic epilepsy, others and brain neoplasms. Others were causes other than those listed in table 1, which each physician was unable to categorise. Median seizure durations before treatment were 42 and 60 min in the FPHT and LEV groups, respectively. No significant differences were observed in any of the basic characteristics examined. Basic characteristics in SAS are shown in online supplemental table 1.

10.1136/jnnp-2022-329485.supp1 Supplementary data

**Table 1:** Basic characteristics

Group	FPHT	LEV	P value
**n**	**80**	**93**	
Age, year	65±19	67±16	0.51
Male sex, (%)	57 (71.3)	60 (64.5)	0.32
Height, cm	163.0±9.1	161.5±9.7	0.32
Body weight, kg	60.3±13.1	57.9±12.5	0.22
Previous history of liver disease	3 (3.7)	3 (3.2)	0.87
Serum creatinine level on admission, mg/dL	0.87 (0.7, 1.1)	0.86 (0.6, 1.1)	0.48
Type of status epilepticus			0.48
Tonic clonic seizure	57 (71.3)	72 (77.4)	
Tonic seizure	0 (0)	1 (1.1)	
Repeated partial seizures	10 (12.5)	10 (10.8)	
Absence of seizures	0 (0)	1 (1.1)	
Complex partial seizure	13 (16.3)	9 (9.7)	
Cause of status epilepticus			0.37
Idiopathic seizure	19 (23.8)	15 (16.1)	
Acute stroke	6 (7.5)	12 (12.9)	
Old stroke	22 (27.5)	31 (33.3)	
Acute trauma	2 (2.5)	2 (2.2)	
Old trauma	5 (6.3)	1 (1.1)	
Brain neoplasm	11 (13.8)	12 (12.9)	
Others	15 (18.8)	20 (21.5)	
Seizure duration before treatment, min	42.0 (21, 90)	60 (30, 80)	0.25

Others were causes other than those listed, which each physician was unable to categorise. Continuous variables with a normal distribution are expressed as the mean±SD and compared using the Student’s t-test. Non-parametric continuous variables are expressed as medians (IQRs) and compared using the Mann-Whitney U test. Categorical variables are expressed as n (%) and compared using the χ^2^ test.

FPHT, fosphenytoin; LEV, levetiracetam.

Others were causes other than those listed, which each physician was unable to categorise. Continuous variables with a normal distribution are expressed as the mean±SD and compared using the Student’s t-test. Non-parametric continuous variables are expressed as medians (IQRs) and compared using the Mann-Whitney U test. Categorical variables are expressed as n (%) and compared using the χ^2^ test.

Treatments are shown in table 2. A median of 10 mg of diazepam was administered before the study drugs in both groups, and the median time between intravenous diazepam and intravenous study drugs was 14 min, without significant differences between the groups. As the study drugs, median doses of 1350 mg FPHT and 2000 mg LEV were administered after diazepam. The total doses of FPHT and LEV administered within 24 hours were 1350 mg and 3000 mg, respectively.

**Table 2:** Treatments for status epilepticus

Group	FPHT	LEV	P value
**n**	**80**	**93**	
Study drug dose administered at loading, mg	1350 (1125, 1500)	2000 (1000, 3000)	
Diazepam dose administered before the study drug, mg	10 (5, 10)	10 (5, 10)	0.35
Time between intravenous diazepam and intravenous study drugs, min	14 (7, 24)	14 (9, 29)	0.61
Study drug dose administered within 24 hours	1350 (1125, 1500)	3000 (1500, 3500)	

Each variable is expressed as a median (IQR) and compared using the Mann-Whitney U test.

FPHT, fosphenytoin; LEV, levetiracetam.

As the primary outcome, seizure cessation rates within 30 min from study drug administration were 83.8% (67/80) in the FPHT group and 89.2% (83/93) in the LEV group (table 3). The rate difference was 5.5% (95% CI −4.7 to 15.7, p=0.29). The non-inferiority of LEV to FPHT was confirmed with p<0.001 by the Farrington-Manning test with a non-inferiority margin of 20%. Regarding the secondary outcome, seizure recurrence rates within 24 hours were 15% in the FPHT group and 17.2% in the LEV group (p=0.70), while intubation rates within 24 hours were 15.0% in the FPHT group and 16.1% in the LEV group (p=0.84), without a significant difference. Regarding the modified Rankin Scale 7 days after admission, 46.3% of patients in the FPHT group and 42.0% in the LEV group had a score of 0 (no symptoms) or 1 (no significant disability) and three patients in the FPHT group and two in the LEV group died. No significant differences were observed between the groups.

**Table 3:** Outcomes

Group	FPHT	LEV	P value
**n**	**80**	**93**	
Primary outcome			
Seizure cessation within 30 min, n (%)	67 (83.8)	83 (89.2)	0.29
Secondary outcome			
Seizure recurrence rate within 24 hours, n (%)	12 (15.0)	16 (17.2)	0.70
Intubation within 24 hours, (%)	12 (15.0)	15 (16.1)	0.84
Other outcomes			
Modified Rankin Scale 7 days after admission			0.74
0 (no symptoms)	21 (26.3)	26 (28.0)	
1 (no significant disability)	16 (20.0)	13 (14.0)	
2 (slight disability)	8 (10.0)	6 (6.5)	
3 (moderate disability)	7 (8.8)	8 (8.6)	
4 (moderately severe disability)	14 (17.5)	18 (19.4)	
5 (severe disability)	11 (13.8)	20 (21.5)	
6 (dead)	3 (3.8)	2 (2.2)	

Each variable is expressed as n (%) and compared using the χ^2^ test.

FPHT, fosphenytoin; LEV, levetiracetam.

The occurrence of serious adverse events related to the study drugs is shown in table 4. Serious adverse events within 1 hour of study drug administration developed in three patients (3.7%) in the FPHT group and none (0%) in the LEV group (p=0.061). One adverse event was cardiac arrest, which is classified as Grade 4 in the Common Terminology Criteria for Adverse Events. This patient died and this death was not directly associated with the administration of FPHT. Respiratory arrest and hypotension were detected in one patient each in the FPHT group, which are classified as Grade 3. No serious adverse events were reported in the LEV group. Furthermore, serious adverse events within 24 hours and 7 days were not reported in either group.

**Table 4:** Serious adverse events related to study drugs

Group	FPHT	LEV	P value
**n**	**82**	**94**	
Serious adverse event within 1 hour, n (%)	3 (3.7)	0 (0)	0.061
Cardiac arrest	1 (1.2)	0 (0)	
Respiratory arrest	1 (1.2)	0 (0)	
Hypertension	1 (1.2)	0 (0)	
Serious adverse event within 24 hours, n (%)	0 (0)	0 (0)	
Serious adverse event within 7 days, n (%)	0 (0)	0 (0)	

Each variable is expressed as n (%) and compared using the χ^2^ test.

FPHT, fosphenytoin; LEV, levetiracetam.

## Discussion

We compared the efficacies of intravenous LEV and intravenous FPHT as second-line treatments following the administration of diazepam for adult convulsive SE with a non-inferiority RCT. Similar efficacies for seizure cessation within 30 min were observed. No significant differences were noted in other efficacies and safety; however, serious adverse events potentially related to the study drugs were only detected in the FPHT group.

This is the first study to confirm the non-inferior efficacy of LEV for the treatment of adult SE after the administration of diazepam in comparisons with FPHT. LEV has frequently been compared with phenytoin as a second-line treatment for SE.^14–19^ However, an RCT has not been performed to confirm the significantly greater efficacy of LEV than that of phenytoin/FPHT and a non-inferiority RCT has yet to be conducted. Therefore, LEV is less frequently recommended than phenytoin/FPHT or not at all in a number of guidelines,^5 30^ and is not covered by the national health insurance systems of many countries for the treatment of SE.^7^ Established Status Epilepticus Treatment Trial (ESETT) recently reported the similar effects of LEV, FPHT and valproate for patients with established SE, including some adults.^19 31^ The main differences between our RCT and ESETT were the targeting of adult SE only and the non-inferiority design. To the best of our knowledge, this is one of the largest RCTs on LEV and FPHT for adult SE with similar efficacy and less serious adverse events of LEV, while other large RCTs included paediatric SE of 2 years of age or older.^17–19 31^

Nevertheless, the occasional adverse events of phenytoin/FPHT, such as hypotension, arrhythmia or respiratory/circulatory arrest, need to be considered in the treatment of SE.^8–10^ Therefore, expert opinions more strongly recommend the use of LEV in the emergency department than phenytoin/FPHT.^13^ Since the present results demonstrated the non-inferiority of the efficacy of LEV without serious adverse events, LEV is recommended as a second-line treatment for SE following benzodiazepine beside phenytoin/FPHT. It is important to note that the present study targeted adult patients with SE, including many elderly patients. Since serious adverse events were previously shown to be more frequent in elderly patients or patients with cardiac diseases treated with phenytoin/FPHT,^10^ LEV may be more strongly recommended for these adult populations.

The present study focused on emergency clinical practice. Under life-threatening conditions, physicians cannot devote time to obtaining informed consent, registry and randomisation. A large RCT of SE was recently performed with the establishment of a system for after-acquired consent.^17 18^ The Clinical Trials act, newly established by the Ministry of Health, Labour and Welfare in Japan in 2017, states that physicians may conduct a study without informed consent under specific conditions, such as emergency and life-threatening situations. The present study is the first to be performed with after-acquired consent in Japan. Furthermore, this study was designed such that physicians easily registered data using smartphone devices. Randomisation and allocation were quickly performed, and dosage/administration and patient information input were guided on their monitor. Since it has become popular worldwide and is permitted by many hospitals, this registration system is considered to be positive and effective for future studies conducted under emergency conditions.

The present study had several limitations. Physicians who treated patients and performed outcome assessments were not blinded. While the dose of phenytoin was 22.5 mg/kg, that of LEV ranged between 1000 mg and 3000 mg according to the Japanese epilepsy guidelines.^26^ The seizure cessation rate within 30 min, the primary outcome of this study, was high in both the LEV and FPHT groups. In contrast to previous studies in which only patients with SE uncontrolled by benzodiazepine were enrolled,^17–19^ we administered second-line drugs after diazepam irrespective of seizure cessation with diazepam. This practice is recommended in the SE guidelines in Japan and other countries,^5 26^ and is the most frequently performed treatment for SE in emergency rooms. Patients whose SE may have stopped with diazepam only may have been included in the patient sample; however, the seizure cessation rate within 24 hours as the secondary outcome was affected by the study drugs because diazepam lost its efficacy within a few minutes. As the other reason, this study was conducted in general emergency and critical care centres, most of which were not the epilepsy centres to which many patients with refractory SE were transported in previous RCTs.^19^ Many elderly patients with SE caused by cerebral strokes were included in this study, which may have affected efficacy and safety. Furthermore, we did not diagnose SE with EEG, similar to previous RCTs on SE.^15–19^ Epilepsy-mimicking diseases, such as psychiatric paroxysmal attacks, were included in the present study. We need to simultaneously exclude non-convulsive SE in future studies. Moreover, treatments other than the study drugs were not defined. Since each physician selected the doses of diazepam and the study drugs, there may have been unknown confounding factors.

## Conclusion

The efficacy of LEV was similar to that of FPHT for the treatment of adult convulsive SE following the administration of diazepam. LEV has potential as a second-line treatment for adult SE.

## Data Availability

Data are available upon reasonable request. The data sets generated and/or analysed during the present study are available from the corresponding author upon reasonable request.
