# Supplementary material for: Levetiracetam versus fosphenytoin as a second-line treatment after diazepam for adult convulsive status epilepticus: a multicentre non-inferiority randomised control trial
Source: J Neurol Neurosurg Psychiatry. 2022 Oct 7;94(1):42–8. doi: 10.1136/jnnp-2022-329485 (PMC9763167; doi:10.1136/jnnp-2022-329485)
Supplement: Supplementary data [file jnnp-2022-329485supp001.pdf]

Supplemental Table.1 Basic characteristics in safety analysis set

Group<br>n	FPHT<br>82	LEV<br>94	p<br>value
Age, year	65 ± 19	67 ± 16	0.32
Male sex, (%)	58 (70.7)	60 (63.8)	0.33
Height, cm	163.0 ± 9.2	161.4 ± 9.8	0.26
Body weight, kg	60.4 ± 13.2	57.9 ± 12.4	0.19
Past history of liver disease	3 (3.7)	3 (3.2)	0.87
Serum creatinine on admission, mg/dL	0.87 (0.7, 1,1)	0.86 (0.6, 1.1)	0.55
Type of status epileptocus			0.43
Tonic clonic seizure	58 (70.7)	72 (76.6)	
Tonic seizure	0 (0)	1 (1.1)	
Repeated partial seizure	10 (12.2)	11 (11.7)	
Absence seizure	0 (0)	1 (1.1)	
Complex partial seizure	14 (17.1)	9 (9.6)	
Cause of status epilepticus			0.49
Idiopathic seizure	19 (23.2)	16 (17.0)	
Acute stroke	7 (8.5)	12 (12.8)	
Old stroke	22 (26.8)	31 (33.0)	
Acute trauma	2 (2.4)	2 (2.1)	
Old trauma	5 (6.1)	1 (1.1)	
Brain neoplasm	11 (13.4)	12 (12.8)	
Others	16 (19.5)	20 (21.3)	
Seizure duration before treatment, minutes	42.0 (30, 90)	60 (30, 80)	0.64

Continuous variables with the normal distribution are expressed as the mean ± standard deviation and are compared using Student's t-test. Non-parametric continuous variables are expressed as the median (interquartile range) and are compared using Mann–Whitney U test. Categorical variables are expressed as n (%) and compared using chi-squared tests. FPHT; fosphenytoin, LEV; levetiracetam
